# ROS generation mediates the anti-cancer effects of WZ35 via activating JNK and ER stress apoptotic pathways in gastric cancer

**DOI:** 10.18632/oncotarget.3333

**Published:** 2015-01-21

**Authors:** Peng Zou, Junru Zhang, Yiqun Xia, Karvannan Kanchana, Guilong Guo, Wenbo Chen, Yi Huang, Zhe Wang, Shulin Yang, Guang Liang

**Affiliations:** ^1^ Chemical Biology Research Center, School of Pharmaceutical Sciences, Wenzhou Medical University, Wenzhou Zhejiang, China; ^2^ School of Environmental and Biological Engineering, Nanjing University of Science and Technology, Nanjing, Jiangsu, China; ^3^ Department of Digestive Diseases, the First Affiliated Hospital of Wenzhou Medical University, Wenzhou, Zhejiang, China; ^4^ Department of Oncological Surgery, the First Affiliated Hospital of Wenzhou Medical University, Wenzhou, Zhejiang, China

**Keywords:** Gastric cancer, ROS, Curcumin analog, ER stress, JNK

## Abstract

Gastric cancer is one of the leading causes of cancer mortality in the world, and finding novel agents and strategies for the treatment of advanced gastric cancer is of urgent need. Curcumin is a well-known natural product with anti-cancer ability, but is limited by its poor chemical stability. In this study, an analog of curcumin with high chemical stability, WZ35, was designed and evaluated for its anti-cancer effects and underlying mechanisms against human gastric cancer. WZ35 showed much stronger anti-proliferative effects than curcumin, accompanied by dose-dependent induction of cell cycle arrest and apoptosis in gastric cancer cells. Mechanistically, our data showed that WZ35 induced reactive oxygen species (ROS) production, resulting in the activation of both JNK-mitochondrial and ER stress apoptotic pathways and eventually cell apoptosis in SGC-7901 cells. Blockage of ROS production totally reversed WZ35-induced JNK and ER stress activation as well as cancer cell apoptosis. In vivo, WZ35 showed a significant reduction in SGC-7901 xenograft tumor size in a dose-dependent manner. Taken together, this work provides a novel anticancer candidate for the treatment of gastric cancer, and importantly, reveals that increased ROS generation might be an effective strategy in human gastric cancer treatment.

## INTRODUCTION

Gastric cancer is the fourth most commonly diagnosed cancer and the second leading cause of cancer-related death in the world [1]. This aggressive disease continues to be a major public health issue worldwide. Surgery is the mainly curative treatment for localized gastric cancer. However, even after complete resection, more than half of patients with locally advanced tumors will recur, and fewer than 40% patients survive beyond 3 years [2]. The high risk of relapse after surgery has led to a search for strategies to prevent relapse and to improve survival for gastric cancer patients. Chemotherapy in advanced gastric cancer is an important issue because the majority of patients with gastric cancer develop metastases during the course of their disease [3, 4]. However, severe side effects and complications such as hematological and gastrointestinal toxicities of current anticancer drugs become major problems in the clinical setting, which highlights the urgent need for novel effective and less toxic therapeutic approaches [5, 6].

Curcumin, a yellow compound isolated from the rhizome of the herb Curcuma longa L, has been demonstrated as a multifunctional bioactive natural product [7]. It also displays great potential as a chemopreventive and therapeutic agent due to its ability to negatively modulate cancer-related biomarkers and inhibit the proliferation of tumor cells but retain pharmacological safety profile *in vivo* [8-10]. Various signaling pathways and molecular targets have been reported to be involved in the anti-cancer effects of curcumin [11, 12]. However, clinical studies have shown that curcumin is less efficacious in human because over 80% of this compound does not reach systemic circulation, but rather is rapidly excreted [13]. In an attempt to retain curcumin's favorable medicinal properties and safety profile while increase its potency, chemical modifications on curcumin have been paid much attentions [14]. Previously, our lab designed and synthesized a several mono-carbonyl analogs of curcumin (MACs) via deletion of β-diketone moiety, and we have demonstrated that these MACs not only enhanced the chemical stability *in vitro* but also significantly improved pharmacokinetic profiles *in vivo* [15]. Then, anti-cancer bio-screenings have been performed on these MACs, among which, a new compound, 1-(4-hydroxy-3-methoxyphenyl)-5-(2-nitrophenyl)penta-1,4-dien-3-one (WZ35), showed particular anti-cancer potency against human gastric cancer and was chosen to evaluate the underlying mechanisms.

Here, our observations demonstrated that chemically stable WZ35 can induce G2/M phase arrest and cell apoptosis in gastric cancer cells, via activating ROS-dependent ER stress and JNK mitochondrial pathways, blockage of ROS production by specific inhibitor totally abolished the anti-cancer effects of WZ35. WZ35 also exhibited good anticancer ability *in vivo*. These studies suggest that WZ35 could be a potential candidate for the treatment of gastric cancer.

## RESULTS

### WZ35 was more stable than curcumin *in vitro*

**Figure 1:**
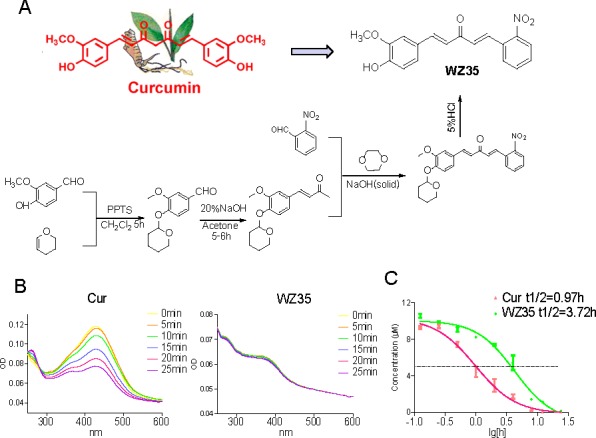
Design, synthesis and chemical stability study of WZ35 (A) The structures of curcumin and WZ35 as well as synthesis scheme of WZ35. (B) UV-visible absorption spectrum of curcumin and WZ35 in phosphate buffer (pH 7.4). (C) Time-concentration curves of WZ35 and curcumin in the culture medium. Using the HPLC methods, the chromatographic peaks of WZ35 and curcumin were identified. Concentration of WZ35 and curcumin in cultural medium was detected by HPLC methods.

The structure and synthesis of WZ35 are shown in Figure 1A. Due to the deletion of the β-diketone moiety in the molecular structure, we hypothesize that WZ35 may possess a more stable structure *in vitro*. Firstly, we tested the chemical stability of WZ35 and curcumin in phosphate buffer (pH 7.4) using absorption spectrum assay. As shown in Figure 1B, the UV-visible absorption spectrum of curcumin displayed an intense peak with an absorption maximum close to 425 nm, and the absorption intensity of curcumin spectra decreases significantly in phosphate buffer (pH 7.4) with time. After 25-minute incubation in the phosphate buffer, curcumin lost more than 45% of its original intensity, while WZ35 showed no degradation under the same condition (Figure 1B). Then, we tested the levels of WZ35 and curcumin in cell cultural medium using the HPLC method to compare their stability in medium. Figure 1C showed the concentration-time profiles of WZ35 and curcumin in cultural medium. After a 4-hour incubation period, curcumin underwent rapid degradation in the medium and more than 90% of curcumin degraded, while more than 50% of the original WZ35 content was retained in the medium. These data suggested that a chemical modification in curcumin attenuated its degradation *in vitro*.

### WZ35 effectively suppressed the proliferation and invasion of human gastric cancer cells

**Figure 2:**
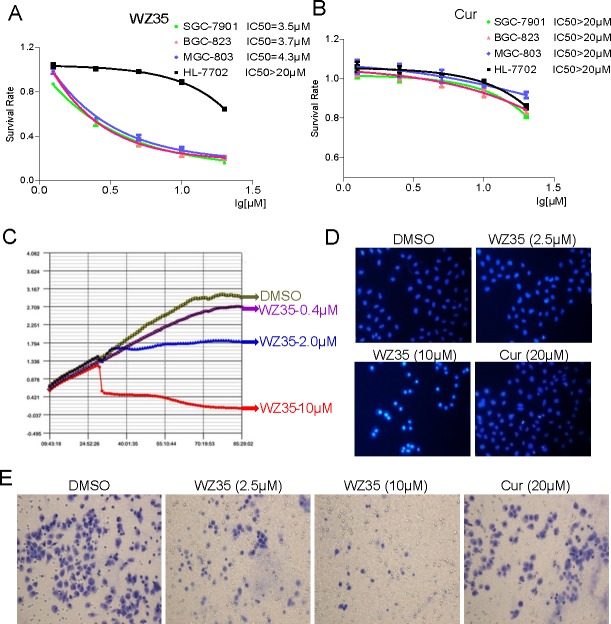
WZ35 inhibits gastric cancer cells growth and migration (A-B) The effects of WZ35 (A) or curcumin (B) on the proliferation of human gastric cancer cells and liver cells. SGC-7901, BGC-823, MGC-803, or HL-7702 cells were incubated with increasing doses of WZ35 or curcumin (1.25-20 μM) for 24h, respectively. Cell viability was determined by MTT assay and the IC_50_ values were calculated. (C) Dynamic monitoring of SGC-7901 cells proliferation. SGC-7901 cells were seeded on laminin-coated plates of an ACEA RT-CES system at a density of 10,000/well and were continuously monitored up to 85 h. At the time point of 30 h, WZ35 at 0.4, 2.0 or 10 μM and vehicle control DMSO were added into the corresponding wells (indicated by color and arrow). (D) WZ35 treatment induced increased apoptotic morphology in MGC-803 cells compared with curcumin. MGC-803 cells were treated with WZ35 (2.5 or 10 μM) or curcumin (20 μM) for 24 h. Cell morphology was observed using an inverted microscope after Hoechst 33258 staining. (E) SGC-7901 cells (10,000/well) and WZ35 (2.5 or 10 μM) or curcumin (20 μM) were added at the same time to the upper compartment of the chamber (pre-coated with diluted Matrigel) in 200 μL serum-free medium, and 500 μL culture medium was added below the chamber. After 24 h of incubation at 37°C, invasive cells stuck to the lower transwell surfaces were fixed and stained with crystal violet, their number quantified with microscopy.

We first determined the effect of WZ35 and curcumin on cell viability of three human gastric cancer cell lines, SGC-7901, BGC-823 and MGC-803, by MTT assay. As show in Figure 2A, treated with WZ35 for 24 h significantly induced cell death in a dose-dependent manner in the three gastric cancer cells with IC_50_ = 3.5, 3.7, and 4.3 μM, respectively. In contrast, only a small percentage of cell death (11.2%) was found in normal human liver HL-7702 cells after treated with 10 μM WZ35 for 24 h. In addition, curcumin at the same concentrations showed slight inhibition against the three gastric cancer cell lines (Figure 2B). To further analyze the anti-proliferative effect of WZ35, we used the RT-CES system to monitor cell number and cell activity in WZ35-treated SGC-7901 cells. As shown in Figure 2C, WZ35 treatment strongly suppressed the proliferation of SGC-7901 cells in a dose-dependent manner. At about 6 h after WZ35 addition, SGC-7901 cells started to undergo death or apoptosis. Further, morphological changes were determined using DAPI staining in MGC-803 cells. Figure 2D revealed that treatment with WZ35 resulted in a concentration dependent increase in the number of apoptotic cells, and WZ35 showed much stronger pro-apoptotic effects than curcumin. Since the invasion ability of cancer cells is critical for cancer development, we next examined the effect of curcumin and WZ35 on inhibition of invasion in SGC-7901 cells. Figure 2E showed that WZ35 treatment significantly reduced the invasion ability of SGC-7901 cells, while curcumin at 20 μM only showed a marginal effect. These results suggest that WZ35, without significant toxicity to normal cells, may possess much more potent anti-cancer ability than curcumin in gastric cancer.

### WZ35 induced G2/M cell cycle arrest in human gastric cancer cells

**Figure 3:**
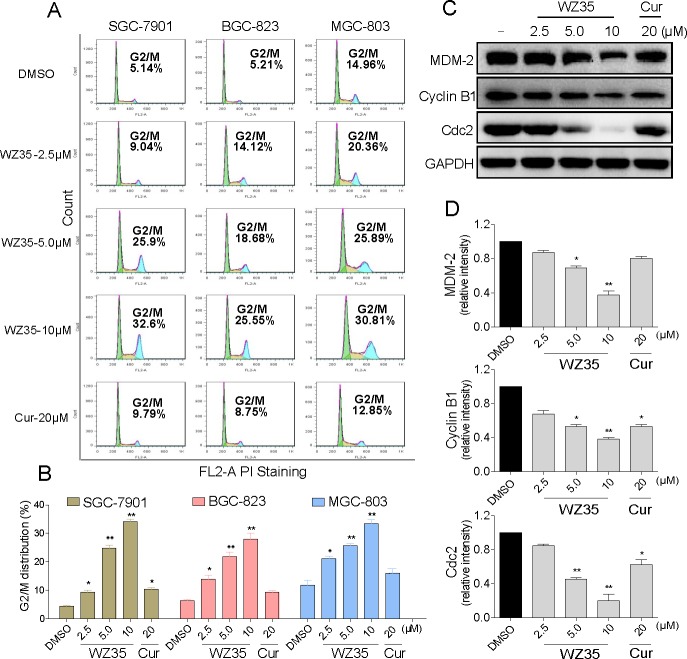
WZ35 induces cell cycle arrest in human gastric cancer cells (A) The three human gastric cancer cells were treated with WZ35 (2.5, 5.0 or 10 μM) or curcumin (20 μM) for 24 h. The number of cells in G2/M phase was determined via flow cytometry. (B) Representative histograms from flow cytometry analysis in the three human gastric cancer cells treated with WZ35 or curcumin. Assays were performed in triplicate. (C) Expression of G2/M cell cycle relative proteins MDM-2, Cyclin B1 and Cdc2 were determined by western blot after treatment with WZ35 (2.5, 5.0 or 10 μM) or curcumin (20 μM) for 24 h. GAPDH was used as internal control. (D) Western blot results from (C) was calculated and represented as the percent of control. (* *p*< 0.05, ** *p* < 0.01).

Three gastric cancer cell lines were treated with WZ35 for 24 h, followed by the cell cycle determination by flow cytometry. The results in Figure 3A and 3B showed that WZ35 dose-dependently induced G2/M arrest in the three cancer cells, while no significant G2/M arrest could be observed in the curcumin-treated group. The western blot analysis revealed that treatment with WZ35 also dose-dependently inhibited the expression of cell cycle-related proteins such as Cyclin B1, MDM-2, and Cdc2 in SGC-7901 cells (Figure 3C and 3D). Consistent with the flow cytometry outcomes, curcumin at 20 μM only showed slight effects on the expression of these proteins. These data suggests that the inhibition of cell proliferation by WZ35 may be partly associated with the induction of G2/M phase arrest.

### WZ35 induced apoptosis in human gastric cancer cells

**Figure 4:**
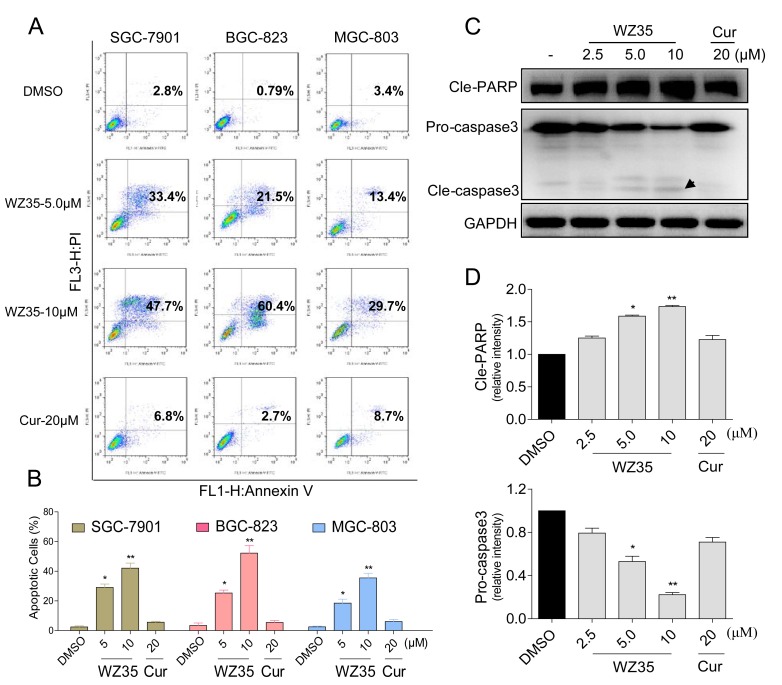
WZ35 induces apoptosis in human gastric cancer cells (A) Induction of apoptosis in human gastric cancer cells was determined by flow cytometry after treatment with WZ35 (5 μM or 10 μM) and curcumin (20 μM) for 24 h. Similar results were obtained in three independent experiments. (B) The percentage of apoptotic cells in the treatment groups was calculated. (C) SGC-7901 cells were treated with WZ35 (2.5, 5 or 10 μM) or curcumin (20 μM) for 24 h. Whole-cell lysates were subjected to western blot to assess the expression of cell apoptosis related proteins. GAPDH was used as internal control. Data represent similar results from three independent experiments. (D) Western blot results from (C) was calculated and represented as the percent of control. (* *p* < 0.05, ** *p* < 0.01).

We further examined the pro-apoptosis effect of WZ35 on human gastric cancer cells using Annexin V/propidium iodide (PI) staining assay. As shown in Figure 4A and 4B, all of three gastric cancer cell lines have shown a concentration-dependent apoptosis after a 24 h treatment with WZ35, while curcumin at 20 μM had no significant effect on these cell lines. Then we determined the levels of apoptosis-related proteins in SGC-7901 cells treated with WZ35. Figure 4C and 4D showed that treatment with WZ35 for 24 h dose-dependently activated caspase-3/PARP pathway and increased the level of cleaved caspase-3/PARP, suggesting that WZ35-induced SGC-7901 cells apoptosis may be associated to caspase-3/PARP pathway activation.

### Both JNK-mitochondrial and ER stress pathways are involved in WZ35-induced apoptosis

**Figure 5:**
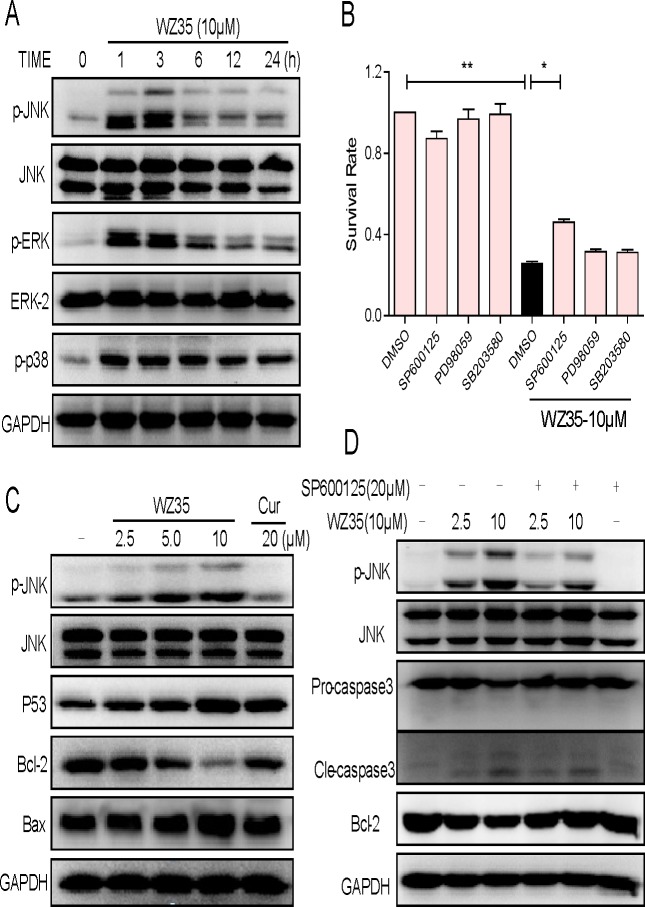
WZ35 activates JNK-mitochondrial apoptotic pathway (A) SGC-7901 cells were treated with WZ35 (10 μM) for the indicated times. The levels of JNK, ERK and p38 phosphorylation were determined by western blot. (B) SGC-7901 cells were pre-incubated with 20 μM JNK inhibitor (SP600125), p38 inhibitor (SB203580) or ERK inhibitor (PD98059) for 1 h before treated with WZ35 (10 μM) for 24 h. Cell viability was determined by MTT assay. (C) SGC-7901 cells were treated with WZ35 (2.5, 5.0 or 10 μM) or curcumin (20 μM) for 1h, the level of JNK phosphorylation was determined by western blot. The p53, Bcl-2 and Bax expression were determined by western blot after treatment with WZ35 or curcumin for 24 h. (D) SGC-7901 cells were treated with WZ35 (2.5 or 10 μM) in the presence or absence of SP600125 (20 μM) for 1 h, JNK phosphorylation was determined by western blot. Bcl-2 and caspase3 inductions were determined by western blot after treatment with WZ35 or curcumin for 24 h. GAPDH was used as internal control. (* p < 0.05, ** p < 0.01).

The next step is to investigate the underlying mechanisms of the anti-cancer effects of WZ35. SGC-7901 cells were used for the subsequent studies. We first found that WZ35 treatment significantly activated all of three pathways of MAPKs, including JNK, ERK, and p38, and their phosphorylation all peaked at approximately 1 h after WZ35 treatment (Figure 5A). We then determined the roles of JNK, ERK, and p38 in WZ35-induced cell apoptosis using specific small-molecule inhibitors. Before treated with WZ35, SGC-7901 cells were pre-treated with JNK inhibitor SP600125, ERK inhibitor PD98059, or p38 inhibitor SB203580, respectively, for 1h. The results in Figure 5B showed that PD98059 or SB203580 alone did not alter the cell viability, but JNK inhibitor SP600125 can partially attenuated WZ35-reduced cell death, indicating that only JNK activation was associated with WZ35-induced cell death. JNK has been well known as a regulator in mitochondrial apoptotic pathway [16]. We therefore examined the effects of WZ35 on Bcl-2 family proteins using western blot assay. As shown in Figure 5C, SGC-7901 cells exposed to WZ35 showed a concentration-dependent reduction of Bcl-2 and a concomitant increase in Bax. In addition, the expression of p53, which has been reported to drive the expression of several proapoptotic proteins such as Bax and Bad [17], was also increased by WZ35 treatment in a dose-dependent manner. In comparison, curcumin at 20 μM only showed slightly effects on Bcl-2 family proteins. Subsequently, SGC-7901 cells were pre-treated with the specific JNK inhibitor (SP600125, 20 μM) for 1 h and then exposed to WZ35 for 1 h. The result in Figure 5D showed that WZ35-induced JNK phosphorylation was effectively reversed by the SP600125, and, the JNK inhibitor also attenuated WZ35-induced increase in cleaved caspase-3 and decrease in Bcl-2 expression. These results suggest that WZ35-induced SGC-7901 cells apoptosis is at least partly mediated by JNK-mitochondrial pathway.

**Figure 6:**
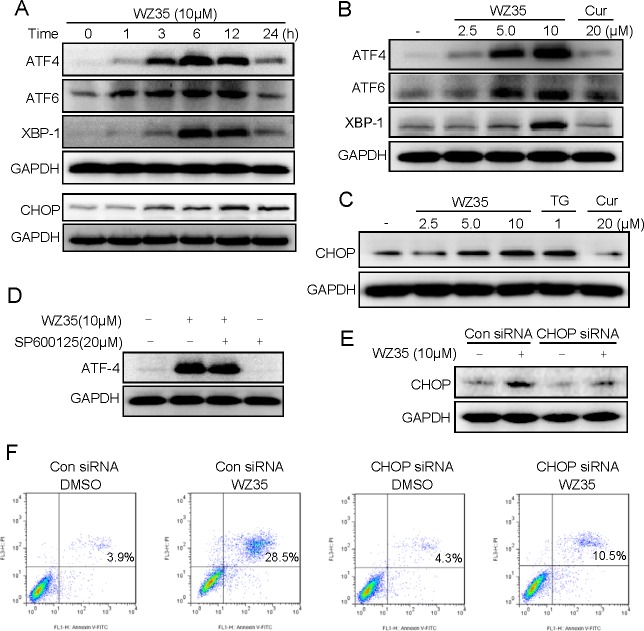
ER stress is involved in WZ35-induced human gastric cancer cells apoptosis (A) SGC-7901 cells were treated with WZ35 (10 μM) for the indicated times, the protein levels of ATF4, ATF6, XBP-1 and CHOP were determined by western blot. (B) SGC-7901 cells were treated with WZ35 (2.5, 5 or 10 μM) or curcumin (20 μM) for 3 h, the ATF4, ATF6 and XBP-1 expression were detected by western blot. (C) The protein level of CHOP was examined by western blot after treatment with WZ35, thapsigargin (TG), or curcumin for 12 h. GAPDH was used as internal control. (D) SGC-7901 cells were pre-incubated with 20 μM JNK inhibitor (SP600125) for 1 h before treated with WZ35 (10 μM), three hours later the ATF4 expression was detected by western blot. (E) SGC-7901 cells were infected with CHOP siRNA or control siRNA, CHOP expression in SGC-7901 cells was determined by western blot after stimulation with WZ35 (10μM) for 12 h. (F) SGC-7901 cells transfected with CHOP siRNA or control siRNA were treated with WZ35 (10 μM) for 24 h. Percentage of cell apoptosis was determined by Annexin-V/PI staining and flow cytometry.

In addition to mitochondrial pathway, ER stress also plays an important role in the initiation of curcumin-induced apoptosis [18]. Thus, we next examined the expressions of ER stress-related proteins, such as transcription factor 4 (ATF4), transcription factor 6 (ATF6), X-box binding proteins 1 (XBP-1), and C/EBP-homologous protein (CHOP) in WZ35-treated SGC-7901 cells. The time-course result indicated that WZ35 (10 μM) could activate ER stress. The protein levels of ATF4, ATF6 and XBP-1 reached the peak at 6 h after treatment, and CHOP peaked after 12 h treatment (Figure 6A). WZ35 also showed dose-dependent activation of these four protein expressions (Figure 6B and 6C). In addition, no significant increase in the expression of ER stress-related proteins could be observed in cells treated with 20 μM curcumin. We then found that JNK blockage by SP600125 could not affect WZ35-induced ATF4 expression, indicating that WZ35-induced ER stress is independent on JNK activation (Figure 6D). CHOP has been considered as a marker of ER stress-induced apoptosis [19]. To further confirm the key role of ER stress in the induction of SGC-7901 cells apoptosis by WZ35, we constructed the siRNA of CHOP gene for silencing CHOP expression in SGC-7901 cells. After transfected with CHOP siRNA, CHOP expression was significantly reduced in WZ35-treated cells compared to the vector-control cells (Figure 6E). Furthermore, Figure 6F showed that when CHOP expression in SGC-7901 cells was silenced, cell apoptosis induced by WZ35 was significantly reduce, indicating that WZ35-induced cell apoptosis is at least partly mediated by ER stress pathway.

### ROS generation is the upstream regulator of WZ35-induced apoptosis

**Figure 7:**
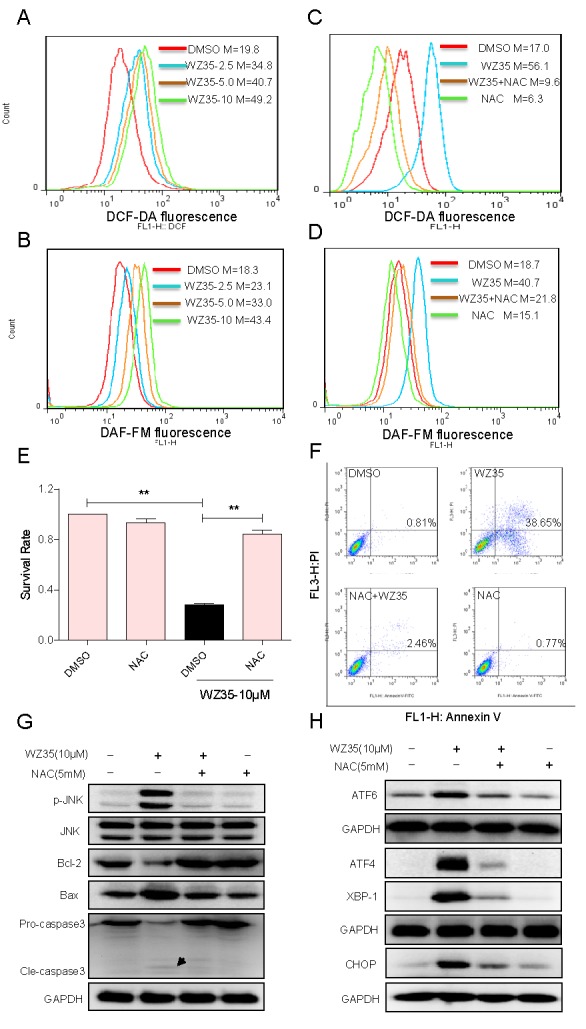
WZ35 induces cytotoxicity in human gastric cancer cells is dependent on intracellular ROS generation (A-B) Intracellular ROS generation induced by increasing doses of WZ35 was measured in SGC-7901 cells by staining with DCFH-DA (10 μM) or 4-amino-5-methylamino-2′,7′-difluorofluorescein (DAF-FM) diacetate (5 μM) and flow cytometry analysis. (C-D) SGC-7901 cells were pre-incubated with 5 mM NAC for 2 h before exposure to WZ35 (10 μM) for 30 min. Intracellular ROS generation was measured by flow cytometry. (E-F) Blocking of ROS generation abolished the cytotoxicity of WZ35. SGC-7901 cells were pre-incubated with or without 5 mM NAC for 2 h before exposure to WZ35 (10 μM) for 24 h. Cell viability was determined by MTT assay (E). Percentage of cell apoptosis was determined by Annexin-V/PI staining and flow cytometry (F). (G-H) SGC-7901 cells were pretreated with or without 5 mM NAC for 2 h before exposure to WZ35, cell lysates were subjected to western blot to analyze the expression of JNK mitochondrial pathway (G), ER stress (H) and apoptosis related factors. GAPDH was used as internal control. Data presented are representative of three independent experiments.

Previous studies have reported that ROS generation could trigger cell apoptosis via activating both mitochondrial and ER stress pathways [20, 21]. Curcumin has been found to increase ROS generation and induce oxidative stress in several cancer cell lines [22, 23]. Therefore, we determined the production of intracellular ROS in WZ35-treated and untreated cells by flow cytometry. As shown in Figure 7A, treatment with WZ35 for 30 min in SGC-7901 cells caused a dose-dependent increase in DCF-reactive ROS. To identify the role of ROS in mediating WZ35's anti-cancer effects, a ROS inhibitor NAC was used, which is commonly used to inhibit ROS production and test ROS inducers, despite some recent studies mentioning that effects of NAC in other pathways [24, 25]. As shown in Figure 7B, pre-treated with the NAC for 2h significantly inhibited the WZ35-induced ROS production. Using a fluorescent probe specific for individual species of ROS, we found that nitric oxide was induced by WZ35 in SGC-7901 cells (Figure 7C). Co-treatment with NAC fully reversed the WZ35-induced increase in nitric oxide (Figure 7D). Interestingly, it was found that NAC almost completely abolished SGC-7901 cells death induced by WZ35 (Figure 7E). Similar results were observed in the cell apoptosis assay detected by flow cytometry (Figure 7F). These results verified that ROS production mediated WZ35-induced cell death. Further, we tested the effect of the ROS inhibitor NAC on WZ35-induced activation of ER stress, JNK/Bcl-2, and caspase-3 cascades in SGC-7901 cells by western blot analysis. As shown in Figure 7G, WZ35-induced changes in JNK phosphorylation, Bcl-2, Bax, and caspase-3 cleavage were all reversed by NAC pretreatment. Also, NAC pretreatment significantly blocked the overexpression of ATF4, ATF6, XBP-1, and CHOP in WZ35-treated SGC-7901 cells (Figure 7H). These results suggest that ROS induction mediates WZ35-activated apoptotic pathways and is critical upstream regulator in WZ35's anti-cancer activity.

### WZ35 inhibits SGC-7901 xenograft tumor growth *in vivo*

**Figure 8:**
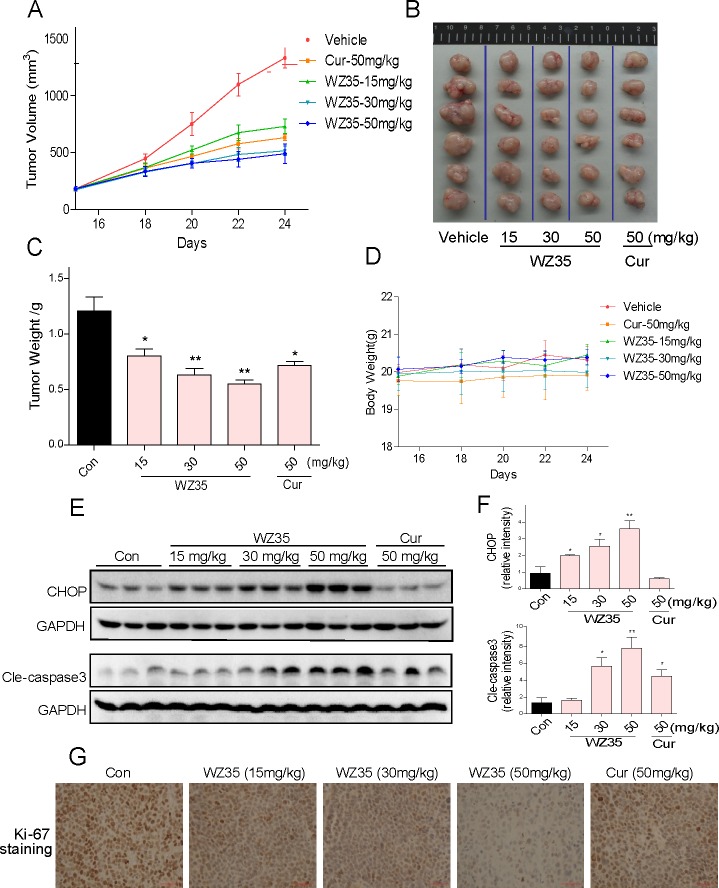
WZ35 inhibits gastric cancer tumor xenograft growth *in vivo* (A) Tumor volumes in WZ35 and curcumin treated mice were smaller than those of vehicle treated mice. SGC-7901 cells were injected to the flanks of nude mice and palpable tumors were allowed to develop for 15 d. Subsequently, nude mice bearing SCG-7901 xenografts received WZ35 orally at the dose of 15, 30 or 50 mg/kg or 50 mg/kg curcumin for a total of 10 doses. Tumor volume was monitored. (B and C) On day 24, tumors were excised and subjected to weight analysis. (D) Body weight of the nude mice in vehicle treated, WZ35 treated and curcumin treated mice. (E-F) Western blot analysis on the expressions of CHOP and caspase3 cleavage from respective tumor tissue lysates. GAPDH was used as protein loading control. Protein levels were quantified by Image-Pro Plus 6.0. (* *p* < 0.05, ** *p* < 0.01). (G) Tumor sections were stained with an anti-Ki-67 Ab to detect proliferating cells.

The *in vivo* anti-tumor effect of WZ35 was evaluated using SGC-7901 tumor xenograft models. The nude mice with SGC-7901 xenografts were treated via oral administration of WZ35 or curcumin once the tumor had grown to a volume of 100-200mm^3^. As shown in Figure 8A-8C, treatment with WZ35 at 15, 30, or 50 mg/kg for 10 days dose-dependently resulted in significant reduction in both tumor volume and weight. Similar results were observed with curcumin at a dosage of 50 mg/kg. Moreover, at the dose of 50 mg/kg, WZ35 displayed better anti-tumor activity than curcumin. In addition, there is no significant difference in body weight change among the vehicle group and WZ35-treated groups, suggesting that WZ35 exhibits no significant toxicity within the 10-day treatment (Figure 8D). Western blot analyses of the tumor tissues revealed that WZ35 treatment increased the levels of CHOP and cleaved caspase-3 in a dose-dependent manner (Figure 8E-8F). Ki-67 staining on tumor tissues showed that Ki-67 expression was inhibited by WZ35 administration in a dose-dependent manner (Figure 8G). Consistent with the tumor size outcomes, WZ35 showed stronger ability in altering CHOP, caspase-3 and Ki-67 profiles in tumor tissues (Figure 8E-8G). These data show that WZ35 exhibits potent anti-tumor activity and high safety *in vivo*.

## DISCUSSION

Various chemotherapy drugs, including doxorubicin, 5-fluorouracil, and cisplatin have been used to treat gastric cancer [26]. Unfortunately, all of these anticancer drugs affect not only pathological tumor cells, but also normal cells [5, 6]. Therefore, the search for new chemopreventive and antitumor agents that are more effective but less toxic has become a matter of great interest.

WZ35 is the one of mono-carbonyl analog of curcumin designed by our group and shows higher chemical stability than curcmin in both PH7.4 phosphate buffer and cell culture medium (Figure 1B and 1C). As a result, a high stability and persistent concentration of WZ35 in the medium may contribute to its better anti-proliferative effects than curcumin at the same concentration (Figure 2). It was then observed that WZ35 significantly induced cell cycle arrest and cell apoptosis in all the three gastric cancer cell lines, accompanied with the corresponding changes in the levels of proteins involved in cell cycle- and apoptosis-related cascades (Figure 2). As expected, curcumin at the concentration of 20 μM showed only a slight effect on both cell cycle arrest and apoptosis. These data suggests that the chemical modification on the natural curcumin is favorable to enhance both stability and pharmacological activity. In the cellular level, our data showed that gastric cancer cells are sensitive to treatment with WZ35 at concentrations ranging from 2.5 to 20 μM, while curcumin did not exhibit good activity at the same concentrations. Importantly, we observed no cytotoxic effects of WZ35 treatment in normal human liver cells, indicating that WZ35, like the leading curcumin, may be safe for use. Besides the cellular effects, we have shown that WZ35 is highly effective at inhibiting tumor growth in a tumor model using nude mice (Figure 8). Also, WZ35 exhibited a high level of safety in mice (Figure 8D).

We then tried to probe the anti-cancer mechanisms of WZ35. After finding the fact that either JNK-mitochondrial or ER stress apoptotic pathway was activated by WZ35 independently and only partly mediated the anti-cancer action of WZ35, we paid attention on the upstream ROS production, which has been reported as a signaling target of curcumin at a high concentration [22]. Under physiological conditions, the maintenance of an appropriate level of intracellular ROS is important in keeping redox balance and cell proliferation [27]. Even a modest increase in ROS levels can stimulate cell growth and proliferation [28]. However, excessive ROS production surmounts cellular antioxidant defenses and triggers apoptosis [29]. Because the increase of ROS in cancer cells may play an important part in the initiation and progression of cancer, such intrinsic oxidative stress is often viewed as an adverse event [30, 31]. Interestingly, cancer cells are more sensitive to rapid increases in ROS levels than normal cells [32]. Cancer cells with increased oxidative stress are likely to be more vulnerable to damage by further ROS insults induced by exogenous agents [33]. Therefore, manipulating ROS levels by redox modulation is a way to selectively kill cancer cells without causing significant toxicity to normal cells [34].

Indeed, redox dysregulation in cancer cells represents a chemical vulnerability that can be targeted by pro-oxidant redox intervention [35]. Overproduced ROS and free radicals lead to serious damage to lipids, proteins, and DNA, and regulate the process involved in the initiation of apoptotic signaling [29, 36]. ROS overproduction could induce the depolarization of the mitochondrial membrane, which eventually results in an increase in the level of other pro-apoptotic molecules in the cytosol [37]. In addition, the role of ER stress in ROS-induced apoptosis has been demonstrated in a variety of cell types [21, 38]. Recently, it has been reported that curcumin-induced apoptosis is due to the production of reactive oxygen species (ROS) [22, 23]. At apoptosis-inducing concentrations, WZ35 induces ROS formation within 30 min of treatment (Figure 7A and 7B). More importantly, blockage of ROS by NAC totally abolished the cytotoxicity and apoptosis induced by WZ35 in SGC-7901 cells. These data validate that WZ35 induces cancer cell death through activating ROS production.

Most of the pharmacological properties of plant polyphenols are considered to reflect their ability to scavenge endogenously generated oxygen radicals or those free radicals formed by xenobiotics, radiations, etc. However, some data in literature suggest that antioxidant properties of the polyphenolic compounds may not fully account for their chemopreventive effects [39]. Husain Yar Khan and his co-workers showed that plant polyphenol induced cell death in human cancer cells involves mobilization of intracellular copper ions and reactive oxygen species generation [40]. Besides, a number of natural and synthetic compounds containing electrophilic Michael acceptor pharmacophores may display promising chemopreventive and chemotherapeutic anti-cancer activity [41]. Prototype Michael acceptors of the α,β-unsaturated aldehyde-class including acrolein, crotonaldehyde, and trans-4-hydroxy-2-nonenal display very high electrophilicity and chemical reactivity that are associated with the interactions with a series of aldo-keto reductase or oxidoreductase with sulfhydryl groups to disrupt the redox balance [42]. Thus, we guess that, the compound WZ35, structurally containing two Michael acceptors (α, β-unsaturated ketone groups), may interact some redox-related enzymes, which contributes to its pro-oxidant and consequent anti-cancer effects. Further studies are necessary to use different ROS inhibitors to comfirm the ROS-mediating mechanism and to definitively identify the direct molecular target of WZ35.

Mitogen-activated protein kinases (MAPKs) are serine-threonine protein kinases that play the major role in signal transduction from the cell surface to the nucleus. Studies have demonstrated that ROS are also involved in the regulation of different signal transduction pathways including MAP kinases and transcription factors [43]. In SGC-7901 cells, treatment with WZ35 for 1-3 h increased the levels of all three pathways in MAPKs. Surprisingly, only JNK activation is involved in the events of WZ35-mediated cell death, which was confirmed by the use of JNK inhibitor SP600125. Considerable evidences suggested that JNK is primarily activated by various environmental stresses including oxidative stress [44]. Figure 7G indicated that ROS blockage completely inhibited WZ35-induced JNK phosphorylation. From these data, we concluded that WZ35-induced apoptosis through ROS-dependent JNK apoptotic pathway. It has been well demonstrated that activation of JNK pathway induces mitochondria-dependent cell apoptosis via activating mitochondrial Bcl-2 family proteins and caspase-3 [16]. We found that WZ35 stimulation significantly increased the level of pro-apoptotic Bax and decreased the level of anti-apoptotic Bcl-2 in SGC-7901 cells, while pretreatment with SP600125 could reverse these changes (Figure 5). Besides, we also found that WZ35-induced activation of Bcl2/Bax pathway was almost completely blocked by NAC (Figure 7G), indicating that ROS acts as a upstream signaling molecules involved in WZ35-induced activation of mitochondrial pathway.

**Figure 9:**
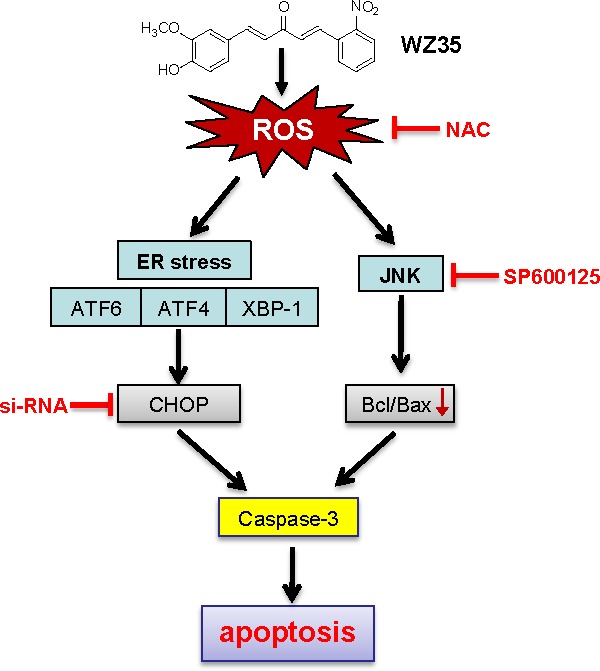
Schematic illustration of the underlying mechanism of WZ35's anti-cancer activity

In addition, in response to oxidative stress mediated by ROS, accumulation of unfolded or misfolded proteins triggers a cellular adaptive procedure known as ER stress [45]. Cells initially adapt to the accumulation of unfolded proteins by inducing the expression of ER-resident molecular chaperones as well as by inhibiting new protein synthesis [46]. However, if this adaptation does not prove sufficient, the apoptotic response is initiated via induction of ER-associated apoptotic molecules. Among the ER-stress related proteins, ATF4, ATF6 and XBP-1 are typical ER stress-regulated proteins involved in ER stress-induced apoptosis [45, 47]. Quite a few studies indicated the regulation of ROS on ER stress activation in a variety of cancer cell lines [21, 48]. Our findings showed ATF4, ATF6 and XBP-1 are induced in a dose-dependent manner after WZ35 stimulation, suggesting that ER stress is activated 3-6 h after WZ35 treatment. These initiations of ER stress apoptotic pathway have been reported to increase CHOP gene expression, triggering ER stress-specific cascade for implementation of apoptosis [49]. Consequently, the up-regulation of CHOP protein expression was observed in SGC-7901 cells after WZ35 treatment for 6-12 h. Then, the question is whether WZ35-induced cell apoptosis is ER stress-dependent or not. CHOP induction is probably most sensitive to ER stress response, and CHOP is considered as a marker of commitment of ER stress-induced apoptosis [19]. Knockdown of CHOP by specific siRNAs also attenuated the desipramine-induced and gamma-tocotrienol-induced ER stress-mediated apoptotic cascade [50, 51]. The data in Figure 6F using CHOP siRNA transfection solidly supports the conclusion that WZ35-induced apoptosis is, at least partially, ER stress-dependent. We found that WZ35-induced activation of ER stress was almost completely blocked by NAC (Figure 7H), indicating that ROS production is also the upstream regulator of WZ35-induced ER stress in gastric cancer cells. Interestingly, we also found that JNK inhibition do not affect WZ35-induced ATF4 expression (Figure 6D), indicating that these two pro-apoptotic pathways do not crosslink and are independently regulated by ROS (Figure 9).

In conclusion, we here investigated the anti-proliferative effects and mechanisms of WZ35, a chemically stable mono-carbonyl analog of curcumin, in gastric cancer cell lines. In this study, we found that WZ35 acts directly as a ROS activator in human gastric cancer cells to induce cytotoxicity in a manner that arrests cell cycle and causes apoptosis. Investigating the mechanism in gastric cancer cells, we found that WZ35 treatment resulted in severe ROS accumulation. Excessive ROS caused the activation of JNK-mitochondrial and ER stress apoptotic pathways. The suppression of apoptosis by NAC validates the critical role of ROS in WZ35-induced cell death. Figure 9 showed a proposed signaling model leading to development of ROS-induced cell death induced by WZ35. These results indicate that the novel curcumin analogue WZ35 possesses great potential as a promising candidate for the treatment of gastric cancer. In addition, we also demonstrated that ROS production could be an important target for the development of new anti-cancer drugs.

## MATERIALS AND METHODS

### Cell culture and reagents

Human gastric cancer cell lines SGC-7901, BGC-823, MGC-803 and normal human liver cell line HL-7702 were purchased from the Institute of Biochemistry and Cell Biology, Chinese Academy of Sciences. The cells were routinely cultured in RPMI 1640 medium (Gibco, Eggenstein, Germany) containing 10% heat-inactivated fetal bovine serum (Hyclone, Logan, UT), 100 units/ml penicillin, and 100 ug/ml streptomycin in a humidified cell incubator with an atmosphere of 5% CO_2_ at 37°C. FITC Annexin V apoptosis Detection Kit I and propidium iodide (PI) were purchased from BD Pharmingen (Franklin Lakes, NJ). Antibodies including anti-Cdc2, anti-XBP-1, anti-Bcl-2, anti-Bax, anti-Cyclin B1, anti-cleaved PARP, anti-MDM-2, anti-caspase-3 p30/17, anti-p53, anti-ATF6, anti-Ki67, anti-GAPDH, goat anti-mouse IgG-HRP, donkey anti-rabbit IgG-HRP and donkey anti-goat IgG-HRP were purchased from Santa Cruz Biotechnology (Santa Cruz, CA). Antibodies including anti-CHOP, anti-ATF4, anti-p-JNK, anti-JNK were purchased from Cell Signaling Technology (Danvers, MA). Matrigel invasion assay Kit was purchased from Corning (Corning Costar, Cambridge, MA).

### Chemistry

Curcumin, SP600125, SB203580, PD98059 and N-acetylcysteine (NAC) were purchased from Sigma (St. Louis, MO). As shown in Figure 1A, curcumin analogue WZ35 was synthesized in our laboratory. The details of synthesis are as follows: The vanilin (0.039 mol) and 3,4-dihydro-2H-pyran (0.197 mol) are dissolved in the dichloromethane (30 ml), PPTS (0.30 mol) was added as a catalyst, and stirred at room temperature for 5 h. After completion of reaction, the solvent was removed by evaporation under reduced pressure and purified by chromatography on silica gel to afford 3-methoxy-4-((tetrahydro-2H-pyran-2-yl)oxy)benzaldehyde. Aqueous sodium hydroxide solution (20% w/v, 5 ml) was added to a solution of 3-methoxy-4-((tetrahydro-2H-pyran-2-yl)oxy)benzaldehyde in acetone (20 ml). The reaction mixture was stirred at room temperature for 5-6 h, then diluted with brine and extracted with ethyl acetate. Finally, (E)-4-(3-methoxy-4-((tetrahydro-2H-pyran-2-yl)oxy)phenyl)but-3-en-2-one (1.81 mmol) and 2-nitrobenzaldehyde (1.99 mmol) are dissolved in mixed suspension (10 ml) which consist of sodium hydroxide powder and dioxane. The reaction mixture was stirred at room temperature for 6 h, then purified by the chromatography on silica gel to afford (1E,4E)-1-(3-methoxy-4-((tetrahydro-2H-pyran-2-yl)oxy)phenyl)-5-(2-nitrophenyl)penta-1,4-dien-3-one. Then 5% HCl (1ml) was added dropwise with stirring, and the end product was filtered and washed with water and dried in vacuum. Before use in biological experiments, compound WZ35 was recrystallized from CH_2_Cl_2_/CH_3_CH_2_OH, and HPLC was used to determine the purity of the compound (98.89%).

### Stability evaluation of WZ35 and curcumin in phosphate buffer

We tested the chemical stability of WZ35 and curcumin in phosphate buffer (pH 7.4) using absorption spectrum assay. Briefly, absorbance readings were taken from 250 to 600 nm using a spectra Max M5 (Molecular Devices, USA). A stock solution of 1 mM curcumin or WZ35 was prepared and diluted with phosphate buffer (pH 7.4) to a final concentration of 20 μM, the absorption spectra were collected for over 25 min at 5 min intervals. The UV-visible absorbance spectrum was measured at 25°C in a 1 cm path-length quartz cuvette.

### Stability of WZ35 and curcumin in cell cultural medium

The SGC-7901 cells in 6-well plate were treated with 10 μM WZ35 and curcumin in medium. After specific intervals of time, the cultural medium was collected, WZ35 and curcumin were extracted by the addition of ethyl acetate, and their concentrations were determined by HPLC method. An Agilent LC (1,200 series) was used for the HPLC analysis. The mobile phase consisted of acetonitrile and water (50/50 to 60/40 v/v in 20 min). Chromatographic separation was obtained using a Beckman C18 reverse-phase column (5 μm, 4.6 mm × 25cm) at room temperature at a flow rate of 1 ml/min. WZ35 and curcumin elution was monitored at a wavelength of 310 nm.

### Cell Viability Assay

Cells were seeded into 96-well plates at a density of 8 × 10^3^ per well and allowed to grow overnight in RPMI 1640 containing 10% heat-inactivated FBS. WZ35 was dissolved in DMSO and diluted with 1640 medium to final concentrations of 1.25, 2.5, 5, 10, and 20μM. The tumor cells were incubated with WZ35 for 24 h before the MTT assay. Curcumin was applied as positive control.

### Dynamic monitoring of SGC-7901 cell proliferation using the RT-CES system

The real-time cell electronic sensing assay is based on electrical impedance readings in cell monolayers plated in wells containing built-in gold electrodes. ACEA RT-CES analyzer, 8-well e-plates, and the integrated software which we have used were from Acea Biosciences Inc (San Diego, CA). Cells were plated at a density of 1 × 10^4^ cells/well in 250 μL of medium. The analyzer and the installed plates were placed in a standard cell culture incubator, at 37°C in a humidified atmosphere of 5% CO_2_. Cells were allowed to adhere to plates overnight. After cells seeded, the analyzer was programmed to take readings during 0-96 h, and WZ35 at 0.4, 2.0 or 10 μM was added to the medium at 28 h after incubation. Data were recorded and analyzed using the integrated software. The cell index is a quantitative measure of the spreading and/or proliferative status of the cells in an electrode-containing well.

### Hoechst 33258 staining

At 24 h after WZ35 (2.5 or 10 μM) or curcumin (20 μM) treatment, cells were fixed, washed twice with PBS and stained with Hoechst 33258 staining solution according to the manufacturer's instructions (Hoechst Staining Kit, Beyotime Biotechnology, China). Apoptotic features of cell death were determined by the staining of cell nuclei with the DNA-binding fluorochrome H33258 assessing chromatin condensation by using inverted fluorescence microscope (Nikon, Japan) with 20X amplification. In each group, five microscopic fields were selected randomly.

### Matrigel invasion assay

SGC-7901 cells matrigel invasion assay was performed in corning transwell insert chambers (8.0-μm pore size) according to the manufacturer's instructions. Briefly, 10,000 cells and test compounds were added at the same time to the upper compartment of the chamber (pre-coated with diluted matrigel) in 200 μL serum-free medium, and 500 μL culture medium was added below the chamber. After 24 h of incubation at 37°C, invasive cells stuck to the lower transwell surfaces were fixed and stained with crystal violet, their number quantified with microscopy (Nikon, Japan) with 20X amplification and expressed as a percentage of the positive control.

### Determination of cellular reactive oxygen species

Cellular ROS contents were measured by flow cytometry. Briefly, 5 × 10^5^ cells were plated on 60-mm dishes, allowed to attach overnight, and exposed to different concentrations of WZ35 for 30 min. Cells were stained with 10 μM DCFH-DA or 5 μM DAF-FM-DA (Beyotime Biotech, Nantong, China) at 37°C for 30 min. Cells were collected and the fluorescence was analyzed using a FACSCalibur flow cytometer (BD Biosciences, CA). In some experiments, cells were pretreated with 5 mM NAC for 2 h prior to WZ35 exposure and analysis of ROS generation.

### Cell apoptosis analysis

SGC-7901, BGC-823 and MGC-803 cells were plated on 60-mm plates for 12 h, and then treated with WZ35 (2.5 or 10 μM) and curcumin (20 μM) for 24 h. Cells were then harvested, washed twice with ice-cold PBS, and evaluated for apoptosis by double staining with FITC conjugated Annexin V and propidium iodide (PI) in binding buffer for 30 min using a FACSCalibur flow cytometer (BD Biosciences, CA)

### Cell Cycle Analysis

SGC-7901, BGC-823 and MGC-803 cells were placed on 60-mm plates for 12 h, and then treated with WZ35 (2.5, 5 or 10 μM) and curcumin (20 μM) for 24 h. Cells were then collected into flow cytometry tubes and centrifuged at 1,000 rpm for 5 min to obtain cell pellets. The supernatant was discarded, and the cells were washed with ice-cold PBS and then re-centrifuged. The cells were resuspended in 100 μL PBS, 3 mL of −20°C ice-cold 75% ethanol was added, and the cells were then incubated for 1 h at −20°C. The cells were washed twice with PBS and 10 mg/mL RNase A was added. Propidium iodide was added to the tubes at a final concentration of 0.05 mg/mL and incubated at 4°C for 20 min in the dark. Cell cycle analysis was performed with FACSCalibur flow cytometer (BD Biosciences, CA).

### Transient transfection of small interfering RNA (siRNA)

The siRNA duplexes used in this study were purchased from Invitrogen (Carlsbad, CA, USA) and have the following sequences: CHOP (NCBI accession no. NM_004083, 5′-GAGCUCUGAUUGACCGAAUGGUGAA-3′). Negative Universal Control (Invitrogen) was used as the control. SGC-7901 cells (3 × 10^5^/well) were seeded into 6-well plates and cultured for 24 h, and then were transfected with siRNA duplexes against human CHOP (100 nM) or control siRNA by lipofectamine 2000 (Invitrogen) according to manufacturer's protocol. Cells were further incubated for 48 h before harvest for detection of CHOP expression by Western blot.

### Western blot analysis

Cells or tumor tissues were homogenized in protein lysate buffer, and debris was removed by centrifugation at 12,000 rpm for 10 min at 4°C. The protein concentrations in all samples were determined by using the Bradford protein assay kit (Bio-Rad, Hercules, CA). After addition of sample loading buffer, protein samples were electrophoresed and then transferred to poly-vinylidene difluoride transfer membranes. The blots were blocked for 2 h at room temperature with fresh 5% nonfat milk in TBST and then incubated with specific primary antibody in TBST overnight at 4°C. Following three washes with TBST, the blots were incubated with horseradish peroxidase-conjugated secondary antibodies for 1 h, and the immunoreactive bands were visualized by using ECL kit (Bio-Rad, Hercules, CA). The density of the immunoreactive bands was analyzed using Image J computer software (National Institute of Health, MD).

### *In vivo* antitumor study

All animal experiments were complied with the Wenzhou Medical University's Policy on the Care and Use of Laboratory Animals. Protocols for animal studies were approved by the Wenzhou Medical College Animal Policy and Welfare Committee (Approved documents: 2012/APWC/0216). Five-week-old athymic BALB/cA nu/nu female mice (18-22 g) purchased from Vital River Laboratories (Beijing, China) were used for *in vivo* experiments. Animals were housed at a constant room temperature with a 12 h:12 h light/dark cycle and fed a standard rodent diet and water. SGC-7901 cells were harvested and injected subcutaneously into the right flank (1 ×10^7^ cells in 150 μL of PBS). When tumors reach a volume of 100-200 mm^3^, mice were administered once daily with WZ35 (orally, 15, 30 or 50 mg/kg) or curcumin (orally, 50 mg/kg). The tumor volumes were determined by measuring length (l) and width (w) and calculating volume (V = 0.5 × l × w^2^) at the indicated time points. At the end of treatment, the animals were sacrificed, and the tumors were removed and weighed for use in histology and proteins expression studies.

### Immunohistochemistry

The harvested tumor tissues were fixed in 10% formalin at room temperature, processed and embedded in paraffin. Parraffin-embedded tissues were sectioned (5 μm thick). Tissue sections were primarily stained with indicated antibodies. The signal was detected by biotinylated secondary antibodies, and developed in DAB. Quantity assay of the immunochemistry data was obtained with Image-Pro Plus 6.0 (Media Cybernetics, Inc, Bethesda, MD).

### Statistical analysis

All experiments were assayed in triplicate (n = 3). Data are expressed as means ± SEM. All statistical analyses were performed using GraphPad Pro. Prism 5.0 (GraphPad, SanDiego, CA). Student's t-test and two-way ANOVA were employed to analyze the differences between sets of data. A *p* value <0.05 was considered statistically significant.
